# Epibenthic predators control mobile macrofauna associated with a foundation species in a subarctic subtidal community

**DOI:** 10.1002/ece3.5570

**Published:** 2019-08-14

**Authors:** Eugeniy Yakovis, Anna Artemieva

**Affiliations:** ^1^ Invertebrate Zoology Department Saint Petersburg State University Saint Petersburg Russia

**Keywords:** barnacles, community structure, crabs, crustacea, facilitation, foundation species, high‐latitude systems, mobile invertebrates, predation, shrimp

## Abstract

Foundation species (FS) are strong facilitators providing habitat for numerous dependent organisms. The communities shaped by FS are commonly structured by interplay of facilitation and consumer control. Predators or grazers often indirectly determine community structure eliminating either FS or their principal competitors. Alternatively, they can prey on the dependent taxa directly, which is generally buffered by FS via forming complex habitats with numerous refuges. The latter case has been never investigated at high latitudes, where consumer control is widely considered weak. We manipulated the presence of common epibenthic crustacean predators to assess their effect on mobile macrofauna of the clusters developed by a FS (barnacle *Balanus crenatus* and its empty tests) in the White Sea shallow subtidal (65° N). While predation pressure on the FS itself here is low, the direct effects of a spider crab *Hyas araneus* and a shrimp *Spirontocaris phippsii* on the associated assemblages were unexpectedly strong. Removing the predators did not change species diversity, but tripled total abundance and altered multivariate community structure specifically increasing the numbers of amphipods, isopods (only affected by shrimp), and bivalves. Consumer control in the communities shaped by FS may not strictly follow the latitudinal predation gradient rule.

## Graphical abstract

Although consumer control strength is generally supposed to decrease with latitude, this trend is not clearly evident for the communities shaped by foundation species. We performed field caging experiments revealing surprisingly strong effect of crabs and shrimp on abundances of mobile organisms associated with barnacle clusters in the White Sea (at 65° N).

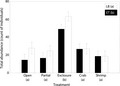

## 1 INTRODUCTION

Consumer control and competition have been long regarded principal drivers of community structure. Emerging evidence that facilitation is at least an equally important structuring force attracted the attention of ecologists to foundation species (hereafter “FS”), strong facilitators that provide habitat for numerous dependent organisms (Bruno, Stachowicz, & Bertness, 2003). Communities shaped by FS (e.g., forests, coral reefs, or seaweed beds) commonly define marine and terrestrial landscapes around the globe. Species abundance and diversity patterns in these communities are obviously determined by facilitation, but also frequently result from interplay of facilitation and consumer control. First, keystone consumers commonly feed on FS, such as sea urchins on kelp or a starfish on corals (Estes & Palmisano, 1974; Kayal et al., 2012). Second, consumers can target principal competitors of FS, such as grazing fishes which keep coral reefs from overgrowth by seaweeds (Hughes et al., 2007; Lewis, 1986; Mumby, Dahlgren, Harborne, & Kappel, 2006). Both processes control the abundance of FS, collaterally affecting all the facilitated organisms. Third, consumers often prey directly on dependent taxa. Yet, FS generally mitigate the impact of consumers on the associated organisms by sustaining habitat structure rich in refuges. Consequently, in the latter case predation is often buffered by habitat complexity, the outcome thus being system‐specific and less predictable.

For instance, despite their complex physical structure packed with shelters, coral reefs exhibit pronounced effects of piscivores on fish abundances (Beets, 1997; Boaden & Kingsford, 2015; Caley, 1993; Hixon & Beets, 1993) and recruitment (Almany, 2004; Almany & Webster, 2006). Predation on juvenile fish can be species‐specific (Carr & Hixon, 1995) or nonselective (Eggleston, Lipcius, & Grover, 1997; Heinlein, Stier, & Steele, 2010). Consumer control can also directly affect sessile fauna of mangroves (Schutte & Byers, 2017) and mobile fauna of seaweed forests (Edgar & Aoki, 1993). In contrast, in seagrass beds, where predation and grazing has been extensively explored, their direct effects on associated organisms are highly variable (see Heck & Orth, 2006 for review), species‐specific (Douglass, Duffy, Spivak, & Richardson, 2007; Leber, 1985), and commonly weak (Bell & Westoby, 1986; Hammerschlag‐Peyer, Allgeier, & Layman, 2013; Summerson & Peterson, 1984). Importantly, consumer control in the communities shaped by FS has been mostly studied in tropical and temperate regions.

Barnacles are foundation species providing habitat for multiple dependent taxa on various hard substrates in temperate and polar waters (Barnes, 2000; Gil & Pfaller, 2016; Harley, 2006; Thompson, Wilson, Tobin, Hill, & Hawkins, 1996; Yakovis, Artemieva, Fokin, Grishankov, & Shunatova, 2005). Aggregated barnacles commonly form two distinct microhabitats types, composed, respectively, by live individuals and their empty tests remaining attached to a subtrate. Empty tests lack the activities of live barnacles such as filter‐feeding and feces production, but contain more cavities and accumulate more sediment, often hosting the macrobenthic assemblages different from those associated with live barnacles (Barnes, 2000; Harley & O'Riley, 2011; Yakovis & Artemieva, 2015). The effect of predators on the suite of species facilitated by barnacles has never been experimentally investigated.

In the White Sea (65° N) shallow subtidal near Solovetsky Islands, a barnacle *Balanus crenatus* is a dominant FS developing clusters on mollusk shells and gravel scattered on muddy sand. Here, clustered barnacles and their comparably numerous empty tests cover all the small hard substrates and host a remarkably diverse assemblage of mobile and sessile macrobenthic taxa (Yakovis & Artemieva, 2017; Yakovis et al., 2005; Yakovis, Artemieva, Shunatova, & Varfolomeeva, 2008), distinctly dissimilar to the fauna of the surrounding unstructured sediment (Yakovis et al., 2005). Equally complex patches of live barnacles and their empty tests develop strongly different assemblages of associated species, with live barnacles accommodating several times more juvenile bivalves *Musculus discors* and polychaetes *Cirratulis cirratus* and *Pygospio elegans* than empty tests (Yakovis & Artemieva, 2015). Also, field experiments with barnacle mimics show that species composition of mobile fauna inhabiting barnacle clusters is largely determined solely by the presence of structurally complex habitat (Yakovis, Artemieva, Fokin, Varfolomeeva, & Shunatova, 2007), potentially rich in refuges from predation.

While spatial distribution of barnacles themselves in tropical and temperate waters is often controlled by predators (Foster, 1987), this does not apparently happen in the White Sea subtidal. Although principal sources of barnacle mortality here are not completely clear (Varfolomeeva, Artemieva, Shunatova, & Yakovis, 2008), their abundance seems rather affected by hard substrate availability (Yakovis, Artemieva, Fokin, Varfolomeeva, & Shunatova, 2013) than controlled by relatively scarce and ineffective consumers (Yakovis & Artemieva, 2015). Weak predation pressure at 65° N is consistent with *biotic interaction hypothesis* (BIH) predicting low importance of biotic interactions (i.e., predation) in subpolar and polar regions (Freestone, Osman, Ruiz, & Torchin, 2011; Schemske, Mittelbach, Cornell, Sobel, & Roy, 2009). BIH has been developed in efforts to explain the latitudinal diversity gradient, which is the strongest global biogeographic pattern observed, and implies that primary selective pressures are coevolution of interacting species in tropics and abiotic factors closer to poles. Although most data available generally confirm BIH, there are multiple cases contradicting one, and predation in subpolar and polar regions is still nearly unexplored compared with temperate and tropical zones (reviewed by Schemske et al., 2009).

Here, we manipulated the presence of common crustacean predators (a shrimp *Spirontocaris phippsii* and a spider crab *Hyas araneus*) to assess their direct effect on mobile macrobenthic assemblages associated with subtidal barnacle clusters in the White Sea. BIH, consistently low predation pressure on the FS itself, and the apparent surplus of potential refuges within barnacle clusters predicted no pronounced effect. At the same time, consumers can strongly affect community structure even in subpolar waters at least indirectly via control of FS abundance (Estes & Palmisano, 1974) or directly in the absence of FS (Quijon & Snelgrove, 2005). In addition, the very presence of FS‐shaped habitat seemingly suggests stronger biotic interactions (i.e., facilitation) than predicted by BIH for subpolar waters.

Given that the microhabitats constituted by live barnacles and their empty tests attract different associated fauna, we used experimental units of both types. This allowed the detection of interactive effects, in case either live barnacles or their empty tests would provide better protection from possible predation. This could, in turn, explain some difference in mobile assemblages between the two microhabitats. Revealing for the first time the effect of predators on FS‐associated dependent assemblage in a subpolar sea would enrich our understanding of interspecific biotic interactions as drivers of community structure and functioning.

## 2 MATERIAL AND METHODS

To test the effect of predators on mobile macrofauna of barnacle clusters, we conducted a year‐long field experiment at a 12‐m‐deep subtidal site in the White Sea near the Solovetsky Islands (the Onega Bay, 65°01.180′N, 35°39.721′E, see Site 1 in Yakovis & Artemieva, 2015). The exposure duration was selected according to the results of previous colonization and caging experiments on the same system (Yakovis & Artemieva, 2015; Yakovis et al., 2007). While caging experiments at lower latitudes typically last shorter, the rates of succession and predation in subtidal of the severely cold White Sea are very slow (Varfolomeeva et al., 2008; Yakovis & Artemieva, 2015; Yakovis et al., 2005) and require longer experiments to detect changes. Also, the accessibility of the study site in late fall and winter is poor due to storms and ice cover. We collected and defaunated (except barnacles and their empty tests with 4+ annual growth rings) empty *Serripes groenlandicus* shells (59 ± 1 mm long) with live barnacles *Balanus crenatus* (hereafter “LB” units), and similar shells with empty barnacle tests (hereafter “ET” units). These units were attached in the alternating order to the bottom of 300 × 375 × 70 mm plastic frames covered with 2.5‐mm nylon mesh. Each frame (hereafter “block”) contained 2 LB and 2 ET units. We randomly distributed these blocks between five treatments: (a) full cages (predator exclosures), (b) open (no mesh, subject to normal predation), (c) partial cages to control for the effect of caging (similar to full cages but with two side windows 175 × 50 mm each), (d) cages with spider crabs *Hyas araneus* added (crab enclosures), and (e) cages with shrimp *Spirontocaris phippsii* added (shrimp enclosures).

Upon retrieval of the blocks, we estimated the wet weight of barnacles in each unit from individual carino‐rostral aperture length measurements according to the previously determined relationship (Yakovis & Artemieva, 2015). We also individually measured empty barnacle tests and calculated their equivalent weight using the same relationship as if they were live. The sum of calculated weights of live and dead barnacles (hereafter “equivalent barnacle weight,” “EBW”) was 40.75 ± 1.27 g per unit (*n* = 124). Neither the number of barnacles or empty tests per unit nor total unit weight could adequately represent habitat capacity because of the variation in size of individual barnacles and empty tests between the units, and lower weight of same sized empty tests compared to live barnacles. We thus selected EBW as a unit size measure to use in further analyses.

Recruitment rate of barnacles at the study site is highly variable between the years (Yakovis et al., 2013). Particularly in 2015–2016, barnacle recruitment was relatively poor, contributing on average 0.5 ± 0.5% to EBW, and the resulting per block EBW in the end of the experiment was similar across Treatment levels (*p* = .433, *F*_4,26_ = 0.984, *n* = 31, one‐way ANOVA, units pooled by block). Mortality of barnacles in LB during the experiment was also low (on average 6.1 ± 1.1% of total EBW per unit) and similar across treatment levels (*p* = .208, *H* = 5.9, Kruskal–Wallis ANOVA, *n* = 31, units pooled by block).

In the field, the crab *Hyas araneus* and the shrimp *Spirontocaris phippsii* both concentrate in epibenthic patches dominated by barnacles and are rarely observed in the surrounding unstructured sediment. Thus, their abundance is rather related to EBW than bottom area. We estimated natural *Hyas* abundance from 45 Petersen grab samples (0.025 m^2^) manually targeted to capture epibenthic patches with barnacles (EBW ≥ 5 g) collected within 100 m from the experimental site in July 1998–1999. There were 0.015 ± 0.004 crabs per gram EBW. This method slightly underestimates the abundance of crabs since the largest individuals can escape the approaching grab. We, however, only used juvenile crabs in the manipulations. Juvenile *Hyas* cannibalize each other within a week (our unpublished laboratory observations); thus, initially we added surplus small crabs to each crab cage to allow self‐thinning. Upon retrieval, there were 0.012 ± 0.002 per gram EBW *Hyas* individuals in crab enclosures (and 0.010 ± 0.004 in partial cages, see Table 1).

**Table 1:** Abundances of crustacean predators by treatment in the field experiment. Zero initial abundances for *Eualis gaimardi* omitted for clarity

Treatment	*Hyas araneus* ≥ 2.5 mm		*Spirontocaris phippsii* ≥ 2.5 mm		*Eualis gaimardi* ≥ 2.5 mm
	Initial	Final	Initial	Final	Final
Open (*n* = 5)					
*N*					
Per block	0	n.a.	0	n.a.	n.a.
Per EBW					
Avg size (mm)		n.a.		n.a.	n.a.
Exclosure (*n* = 8)					
*N*					
Per block	0	0.13 ± 0.13	0	0.13 ± 0.13	0
Per EBW		0.001 ± 0.001		0.001 ± 0.001	
Avg size (mm)		5.4 (*n *= 1)		2.5 (*n *= 1)	
Partial (*n* = 6)					
*N*					
Per block	0	1.67 ± 0.61	0	6.50 ± 0.89	2.33 ± 1.05
Per EBW		0.010 ± 0.004		0.040 ± 0.005	0.015 ± 0.007
Avg size (mm)		12.83 ± 3.26 (*n *= 10)		5.55 ± 0.23 (*n *= 39)	7.93 ± 0.77 (*n *= 14)
Crab (*n* = 6)					
*N*					
Per block	10	1.83 ± 0.31	0	0	0
Per EBW	0.066 ± 0.002	0.012 ± 0.002			
Avg size (mm)	8.88 ± 0.05 (*n *= 60)	17.85 ± 0.18 (*n *= 11)			
Shrimp (*n* = 6)					
*N*					
Per block	0	0	4	3.17 ± 0.31	0
Per EBW			0.025 ± 0.001	0.020 ± 0.002	
Avg size (mm)			5.85 ± 0.18 (*n *= 24)	7.50 ± 0.16 (*n *= 19)	

Highly mobile *Spirontocaris* avoids grabs and corers, and partly escapes hand nets. Consequently, their natural density estimates were much less accurate. The lower estimate was 1.66 ± 0.26 m^−2^ (~0.003 per gram EBW), based on 20 quantitative hand net samples collected within 100 m from the experimental site in July 2012–2015. Yet, the distribution was highly patchy, with most samples containing no shrimp. The higher estimate based on the number of individuals found in six auxiliary partial cages (each exposed for a year in 2009–2012) was 0.11 ± 0.02 per gram EBW. We opted to add 4 adult *Spirontocaris* individuals per shrimp cage, which resulted in the abundance of 0.020 ± 0.002 per gram EBW (compared with 0.040 ± 0.005 in partial cages retrieved in 2016, see Table 1).

The blocks were anchored to the bottom in July 2015 in a haphazard pattern (at least 0.5 m from each other within a square approximately 20 by 20 m) and exposed for 1 year. There was no mesh clogging, and both sedimentation and algal growth rates were slow enough to allow no maintenance along the year. To compensate for intrusion by juvenile shrimp, we deployed extra predator exclosures and excluded from further processing those having (in the end of the experiments) more than one shrimp with carapace length ≥2.5 mm. One open block could not be found in 2016. As a result, we examined 8 predator exclosures, 5 open blocks, and 6 blocks per any other treatment (31 in total). In addition to *Spirontocaris* and *Hyas*, some *Eualis gaimardi* shrimp were found in partial cages by the end of the experiments (Table 1). In the end of the experiments, each block was carefully lifted onboard the vessel in a separate enclosed container, and after that, experimental units were immediately separated to prevent animal movement between the replicates. Each LB and ET unit was then sorted as a separate sample. The sediment between and within the tests of live and dead barnacles was washed out and sieved (0.5 mm mesh). Mobile macrobenthic organisms were identified to lowest possible taxonomic level (generally species or genus) and counted.

To test the effects of manipulations on community structure, we analyzed square root transformed abundances of mobile macrofauna using PERMANOVA (multivariate analysis of variance, Anderson, 2001) based on Bray–Curtis dissimilarities. The factors were Treatment (fixed with five levels corresponding to block types), Live (fixed, ET or LB), Treatment × Live interaction (fixed), Block (random, nested in Treatment), Block (nested in Treatment) × Live interaction (random), and EBW as covariate (fixed). We examined pairwise differences between Treatment levels with the following set of contrasts: (a) open blocks versus partial cages to check for the artifacts of the caging procedure, (b) open blocks and partial cages versus predator exclosures to test for the effect of predation, (c) open blocks and partial cages versus crab cages, (d) predator exclosures versus crab cages to assess the effect of *Hyas*, (e) open blocks and partial cages versus shrimp cages, and (f) predator exclosures versus shrimp cages to assess the effect of *Spirontocaris*. The abundances of *Spirontocaris phippsii* and *Hyas araneus* were excluded from the analysis. To check for homogeneity of variances, we performed PERMDISP test (Anderson, 2006), which indicated that group variances were heterogenous. Given that the largest group (namely predator exclosures) in our slightly unbalanced design had the smallest dispersion, this could potentially cause too liberal results of PERMANOVA test (Anderson & Walsh, 2013). Knowing that balanced PERMANOVA designs are insensitive to variance heterogeneity (Anderson & Walsh, 2013), we assessed the reliability of our results by running ten additional PERMANOVA tests, each on a separate nearly balanced subset of the data with two random exclosure blocks excluded. All the 10 tests produced the results consistent with PERMANOVA on the full data set, confirming the reliability of the latter.

SIMPER procedure (Clarke, 1993) was used to identify the taxa principally responsible for the differences between the factor levels revealed by PERMANOVA. To visualize multivariate differences between the assemblages associated with combinations of Treatment and Live levels, we performed principal coordinates ordination (PCO) based on the Bray–Curtis dissimilarity matrix calculated on square root transformed abundances of taxa divided by EBW. On an additional plot, each combination of levels was represented by a single centroid to increase the clarity (Anderson, Gorley, & Clarke, 2008). We applied cluster analysis (complete linkage) to reveal the relationships between the centroids and used the results to mark distinguished groups on the PCO plot.

Total abundance, species diversity, and abundances of 15 most abundant taxa were analyzed with GLM ANCOVAs using the same design as PERMANOVA above, followed by pairwise comparisons of means between the levels of Treatment and Live with Tukey's HSD *post hoc* tests. Prior to analyses, the response variables were transformed to achieve homogeneity of variances (Cochran's test).

Multivariate community analyses were conducted in PRIMER 6.0 software with PERMANOVA add‐on (Anderson et al., 2008). GLM ANCOVAs were performed in STATISTICA 8.0 software package (Statsoft Inc., 2007). All mean values are given ± *SE*.

## 3 RESULTS

**Figure 1:**
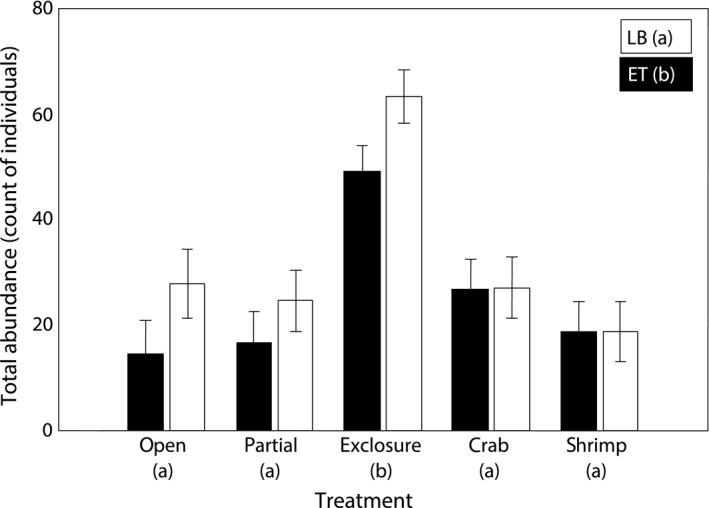
Least squares (LS) mean (± *SE*) total abundances of mobile macrofauna by treatment calculated at EBW = 40.75 in the field experiment (see Table 3 for ANCOVA results). White bars denote live barnacles (LB), and black bars denote empty barnacle tests (ET). Letters in parenthesis indicate homogeneous groups according to Tukey's HSD *post hoc* tests for the effects Treatment and Live (Table 3)

In total, within the experimental units we identified 74 taxa of mobile macrofauna, including 40 polychaete, 9 gastropod, 8 amphipod, and 7 bivalve species, not counting *Hyas araneus* and *Spirontocaris phippsii*. Total abundance of mobile macrofauna was affected both by Treatment and Live (Table 2) being significantly (2.4–3.1 times) higher in predator exclosures compared with any other treatment and 1.4 times higher in LB compared with ET (Table 3 and Figure 1). Treatment × Live interaction was insignificant. Number of taxa was only affected by Treatment (Table 2), being significantly higher in predator exclosures than in any other treatment (Table 3). Species diversity was slightly (but significantly) higher in LB than ET with no significant effect of Treatment (Tables 2 and 3).

**Table 2:** Sum of squares (SS) values from ANCOVA on abundances of 15 most abundant taxa, total abundance, number of taxa, and log‐e species diversity (*H*′) in the field experiment

Higher taxon	Source of variation	Treatment	Live	Tr × Li	EBW	Block(Tr)	Li × Bl(Tr)	Error
	*df*	4	1	4	1	26	26	61
	Species (transformation)	Fixed	Fixed	Fixed	Fixed	Random	Random	
Polychaeta	*Ophelina acuminata* (frt)	**4.50** *	**4.22** ***	**2.77** **	0.16	**9.30** **	2.82	11.82
Polychaeta	*Amphitrite cirrata* (frt)	2.99	1.26	1.11	**1.27** *	**21.25** *	8.35	11.93
Polychaeta	*Capitella capitata* (sqrt)	1.95	0.34	1.84	0.80	13.15	9.51	18.56
Polychaeta	*Chone* sp. (sqrt)	2.93	3.22	6.27	**4.81** **	39.37	**29.60** *	36.24
Polychaeta	*Cirratulus cirratus* (sqrt)	2.17	**12.74** ***	1.28	**15.23** ***	17.84	11.80	36.00
Polychaeta	*Harmothoe imbricata* (none)	1.67	0.18	1.71	2.46	11.56	13.37	44.71
Polychaeta	*Pholoe minuta* (sqrt)	2.19	0.01	**4.32** *	**3.05** **	10.47	9.89	22.38
Polychaeta	*Polycirrus medusa* (sqrt)	1.64	**5.26** ***	1.19	0.06	7.33	4.85	9.31
Polychaeta	*Pygospio elegans* (sqrt)	1.47	**10.39** **	3.17	**6.83** **	42.85	28.20	40.78
Polychaeta	*Sphaerosyllis erinaceus* (sqrt)	**8.60** *	0.41	1.60	**4.37** ***	16.28	7.00	21.86
Bivalvia	*Hiatella arctica* (sqrt)	**26.15** ***	0.48	0.96	0.15	5.20	6.77	16.54
Bivalvia	*Musculus discors* (frt)	**20.51** ***	**2.44** **	2.49	**3.90** ***	12.17	7.82	14.37
Isopoda	*Munna* sp. (frt)	**12.67** **	0.53	0.32	0.13	17.32	11.53	19.13
Amphipoda	*Crassicorophium bonellii* (frt)	2.80	0.14	2.24	1.42	**22.89** **	7.14	27.25
Amphipoda	*Dyopedos porrectus* (frt)	**21.68** **	0.11	1.88	**0.90** *	**29.87** *	**13.20** ***	11.74
	Total abundance (frt)	**8.57** ***	**0.68** **	0.37	3.03***	**7.44** ***	2.69	4.52
	Number of taxa (none)	**474.85** **	43.73	44.79	**346.38** ***	**724.15** **	282.68	571.79
	*H*′ (none)	1.33	**0.52** *	1.11	**1.38** **	**6.95** *	2.91	7.64

Note

Significant effects highlighted in bold.

Abbreviations: EBW, equivalent barnacle weight (covariate); frt, fourth root; sqrt, square root (see text for details).

* *p* < .05, **p < .01, ***p < .001.

**Table 3:** Mean (± *SE*) abundances of 15 most abundant taxa, total abundance, number of taxa, and log‐e species diversity (*H*′) by treatment in the field experiment

Species (transformation)	Significant effects	Treatment levels					Live levels	
		Open	Partial	Exclosure	Crab	Shrimp	LB	ET
*Ophelina acuminata* (frt)	Tr, Li, Tr × Li	0.2 ± 0.1 a	0.5 ± 0.2 a	1.4 ± 0.3 b	0.3 ± 0.1 a	0.5 ± 0.2 ab	0.2 ± 0.1	**1.1 ± 0.2**
	LB	0.2 ± 0.1 a	0.0 ± 0.0 a	0.3 ± 0.2 a	0.2 ± 0.1 a	0.3 ± 0.1 a		
	ET	0.2 ± 0.1 a	1.0 ± 0.4 ab	2.5 ± 0.4 b	0.3 ± 0.2 a	0.8 ± 0.3 ab		
*Amphitrite cirrata* (frt)	—	0.7 ± 0.3	1.0 ± 0.4	2.6 ± 0.7	1.5 ± 0.4	1.0 ± 0.4	1.5 ± 0.3	1.4 ± 0.3
*Capitella capitata* (sqrt)	—	1.0 ± 0.3	0.4 ± 0.2	0.5 ± 0.1	0.8 ± 0.3	0.4 ± 0.1	0.6 ± 0.1	0.5 ± 0.1
*Chone* sp. (sqrt)	—	1.8 ± 1.0	1.2 ± 0.5	2.4 ± 0.8	1.0 ± 0.4	1.1 ± 0.4	2.0 ± 0.4	1.1 ± 0.4
*Cirratulus cirratus* (sqrt)	Li	1.3 ± 0.5	1.1 ± 0.5	1.3 ± 0.3	1.8 ± 0.7	1.6 ± 0.5	**2.1 ± 0.3**	0.7 ± 0.3
*Harmothoe imbricata* (none)	—	0.8 ± 0.2	0.6 ± 0.1	0.9 ± 0.2	0.8 ± 0.2	0.7 ± 0.1	0.8 ± 0.1	0.7 ± 0.1
*Pholoe minuta* (sqrt)	Tr × Li	0.7 ± 0.2	0.6 ± 0.2	1.2 ± 0.2	0.5 ± 0.1	0.8 ± 0.2	0.9 ± 0.1	0.7 ± 0.1
	LB	0.7 ± 0.3 a	1.0 ± 0.3 a	1.5 ± 0.4 a	0.5 ± 0.2 a	0.4 ± 0.3 a		
	ET	0.6 ± 0.2 a	0.3 ± 0.1 a	0.9 ± 0.3 a	0.4 ± 0.2 a	1.2 ± 0.3 a		
*Polycirrus medusa* (sqrt)	Li	0.1 ± 0.1	0.3 ± 0.1	0.5 ± 0.1	0.3 ± 0.1	0.3 ± 0.1	**0.6 ± 0.1**	0.0 ± 0.0
*Pygospio elegans* (sqrt)	Li	1.4 ± 0.5	2.3 ± 0.8	1.6 ± 0.4	1.3 ± 0.5	2.5 ± 1.3	**2.5 ± 0.4**	1.1 ± 0.5
*Sphaerosyllis erinaceus* (sqrt)	Tr	0.4 ± 0.2 a	0.6 ± 0.3 a	1.5 ± 0.3 b	0.5 ± 0.1 a	0.8 ± 0.2 ab	0.7 ± 0.2	0.8 ± 0.2
*Hiatella arctica* (sqrt)	Tr	0.6 ± 0.2 **a**	0.4 ± 0.1 **a**	2.3 ± 0.2 **b**	0.3 ± 0.1 **a**	0.7 ± 0.1 **a**	1.0 ± 0.1	0.9 ± 0.2
*Musculus discors* (frt)	Tr, Li	3.9 ± 1.1 **bc**	0.5 ± 0.1 **a**	11.1 ± 2.7 **d**	5.2 ± 1.2 **c**	1.3 ± 0.3 **ab**	**7.0 ± 1.5**	2.7 ± 0.6
*Munna *sp. (frt)	Tr	1.4 ± 0.5 **a**	1.0 ± 0.3 **a**	5.5 ± 1.1 **b**	3.9 ± 0.8 **b**	1.0 ± 0.4 **a**	2.7 ± 0.6	2.9 ± 0.5
*Crassicorophium bonellii* (frt)	—	2.9 ± 1.1	6.7 ± 2.2	4.1 ± 1.2	1.5 ± 0.6	2.2 ± 0.8	3.5 ± 0.9	3.5 ± 0.8
*Dyopedos porrectus* (frt)	Tr	2.9 ± 1.1 **a**	1.0 ± 0.2 **a**	15.1 ± 3.3 **b**	2.1 ± 0.5 **a**	1.3 ± 0.4 **a**	6.5 ± 1.8	3.9 ± 0.8
Total abundance (frt)	Tr, Li	23.4 ± 4.5 **a**	21.0 ± 4.5 **a**	56.3 ± 6.6 **b**	25.5 ± 3.3 **a**	18.6 ± 2.8 **a**	**36.2 ± 4.0**	25.4 ± 3.2
Number of taxa (none)	Tr	8.8 ± 1.1 **a**	8.0 ± 0.7 **a**	13.0 ± 0.9 **b**	10.4 ± 0.8 **a**	8.7 ± 0.7 **a**	9.1 ± 0.6	10.9 ± 0.5
*H*′ (none)	Li	1.7 ± 0.1	1.7 ± 0.1	1.9 ± 0.1	1.9 ± 0.1	1.8 ± 0.1	**1.9 ± 0.1**	1.8 ± 0.1

Note

Letters indicate the results of Tukey *post hoc* tests where Tr or Tr × Li are significant. Means for Treatment levels provided separately for LB and ET where the interaction is significant. Significantly higher LB or ET means highlighted in bold.

Abbreviations: ET, empty tests; LB, live barnacles; Li, significant Live effect; Tr × Li, significant interaction; Tr, significant Treatment effect; (see Table 2).

**Figure 2:**
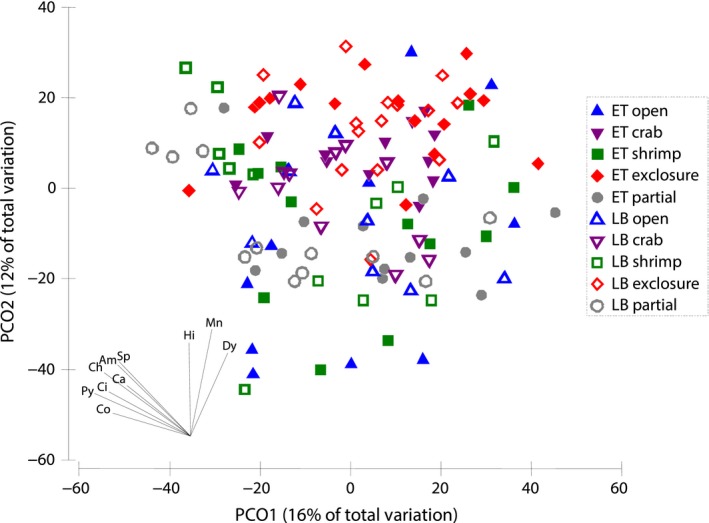
Principal coordinates ordination (PCO) of experimental units based on Bray–Curtis similarities of square root transformed per EBW abundances of mobile taxa. Vectors denote the contributions of specific taxa where correlation (indicated by vector length) exceeds +0.40. Am, *Amphitrite cirrata*; Ca, *Capitella capitata*; Ch, *Chone* sp.; Ci, *Cirratulus cirratus*; Co, *Crassicorophium bonellii*; Dy, *Dyopedos porrectus*; Hi, *Hiatella arctica*; Mn, *Munna *sp.; Po, *Polycirrus medusa*; Py, *Pygospio elegans*; Sp, *Sphaerosyllis erinaceus*

**Figure 3:**
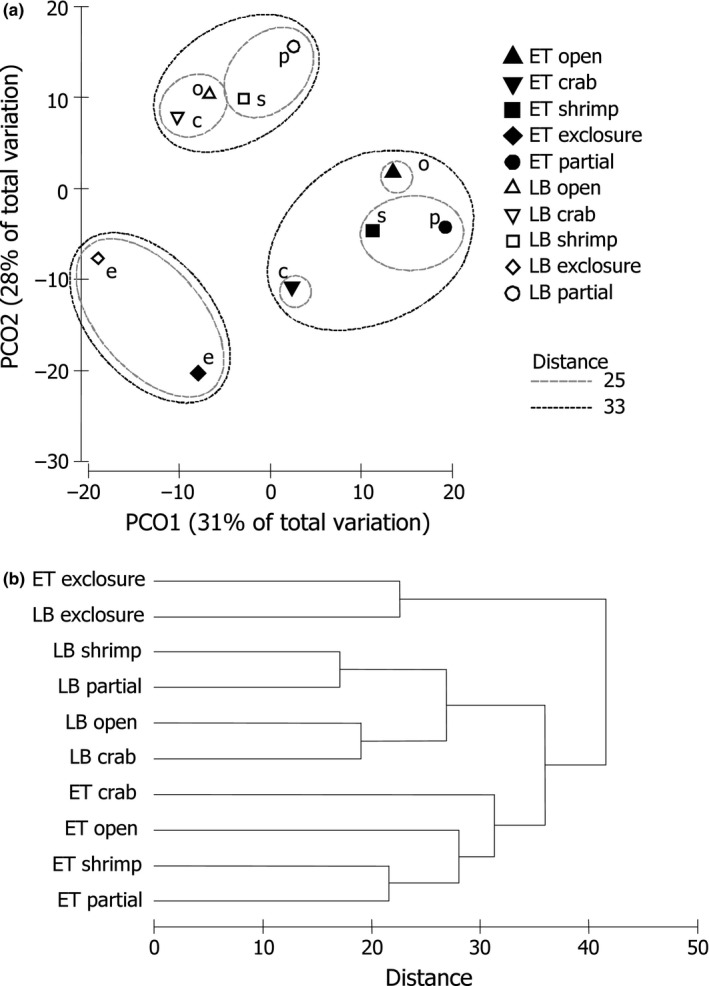
Principal coordinates ordination (PCO) (a) and dendrogram (complete linkage clustering) (b) of distances among centroids on the basis of the Bray–Curtis measures of square root transformed per EBW abundances of mobile taxa. Centroids represent average distances between treatments. For details on differences between treatments, see the results of PERMANOVA in Table 4

Assemblages were significantly affected both by Treatment and Live effects, but not their interaction (see Table 4 for PERMANOVA results). There was no significant difference between open blocks and partial cages, whereas exclosure cages were significantly different from both. Crab and shrimp enclosures were statistically different from exclosures and similar to open blocks and partial cages (Table 4). On the PCO plot, the samples from exclosures also concentrated separately from other treatments with a moderate overlap (Figure 2). According to SIMPER results, abundances of juvenile bivalves *Musculus discors*, amphipods *Dyopedos porrectus*, and isopods *Munna* sp. were primarily responsible for the pairwise dissimilarities between predator exclosures and other treatments (except for the pairwise comparison with partial cages, where *M. discors*, *D. porrectus,* and an amphipod *Crassicorophium bonellii* mostly contributed to the difference). Centroids of assemblages associated with combinations of Treatment and Live levels grouped into three distinct clusters. The first one contained both LB and ET from predator exclosures. LB from all other treatments comprised the second cluster, while ET from all other treatments grouped into the third one (Figure 3).

**Table 4:** Results of PERMANOVA (multivariate analyses of variance) on square root transformed abundances of mobile macrofauna (Bray–Curtis dissimilarities) in the field experiment (see text for details)

Source of variation	*df*	SS	pseudo‐F	p (perm)	Unique perms
EBW (fixed, covariate)	1	4,324	2.6832	**0.002** **	9,942
Treatment (fixed)	4	27,136	2.0136	**0.003** **	9,879
· contrast O versus P	1	4,474	1.0896	0.358	9,924
· contrast O & P versus E	1	15,087	4.7024	**0.001** ***	9,944
· contrast O & P versus C	1	6,068	1.5767	0.112	9,933
· contrast O & P versus S	1	1702	0.3945	0.931	9,918
· contrast E versus S	1	8,950	3.0763	**0.007** **	9,946
· contrast E versus C	1	7,588	3.2583	**0.002** **	9,932
Live (fixed)	1	11,915	5.6546	**0.001** ***	9,934
Tr × Li (fixed)	4	8,877	1.0426	0.405	9,884
Li × Bl(Tr) (random)	26	55,461	1.3237	**0.004** *	9,720
Block(Tr) (random)	26	87,865	2.0971	**0.001** ***	9,742
Error	61	98,302			

Note

*p*‐Values for significant effects and contrasts highlighted in bold.

Abbreviations: C, crab enclosures; E, exclosure cages; EBW, equivalent barnacle weight; O, open blocks; P, partial cages; S, shrimp enclosures.

** *p* < .01, ***p < .001.

**Figure 4:**
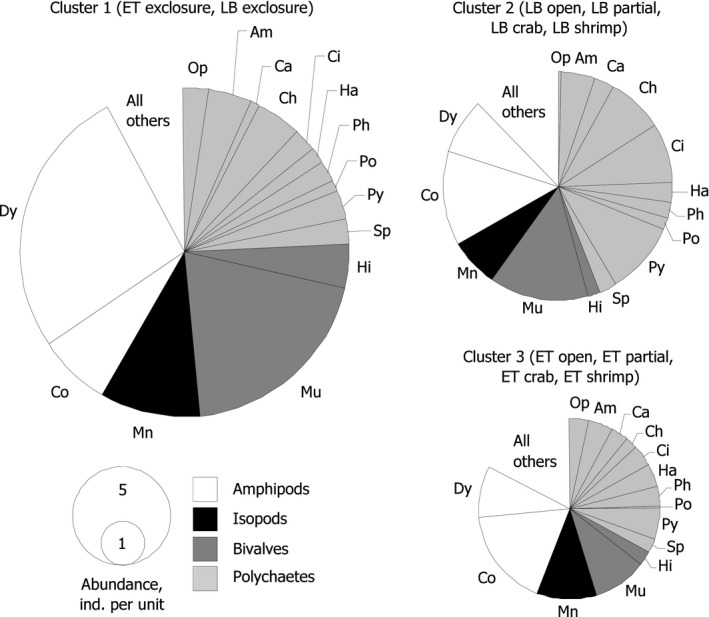
Contributions of 15 most abundant taxa to total abundance by cluster (see Figure 3) comprising centroids on the basis of the Bray–Curtis measures of square root transformed per EBW abundances. Pie chart area denotes average total abundance of mobile macrofauna per experimental unit. Am, *Amphitrite cirrata*; Ca, *Capitella capitata*; Ch, *Chone* sp.; Ci, *Cirratulus cirratus*; Co, *Crassicorophium bonellii*; Dy, *Dyopedos porrectus*; Ha, *Harmothoe imbricate*; Hi, *Hiatella arctica*; Mn, *Munna* sp.; Mu, *Musculus discors*; Op, *Ophelina acuminate*; Ph, *Pholoe minuta*; Po, *Polycirrus medusa*; Py, *Pygospio elegans*; Sp, *Sphaerosyllis erinaceus*

Samples were numerically dominated by juvenile bivalves *Musculus discors*, amphipods *Dyopedos porrectus* and *Crassicorophium bonellii*, isopods *Munna* sp., and polychaetes *Cirratulis cirratus*, *Chone* sp., and *Pygospio elegans* (Figure 4 and Table 3). Total abundance and relative abundance of *Dyopedos porrectus* and juvenile *Musculus discors* were highest in the samples belonging to the first cluster (i.e., LB and ET from predator exclosures; Figure 4). There were relatively more polychaetes in the samples from the second and third clusters (containing, respectively, all other LB and ET), while total abundance in the third cluster was lowest (Figure 4).

**Figure 5:**
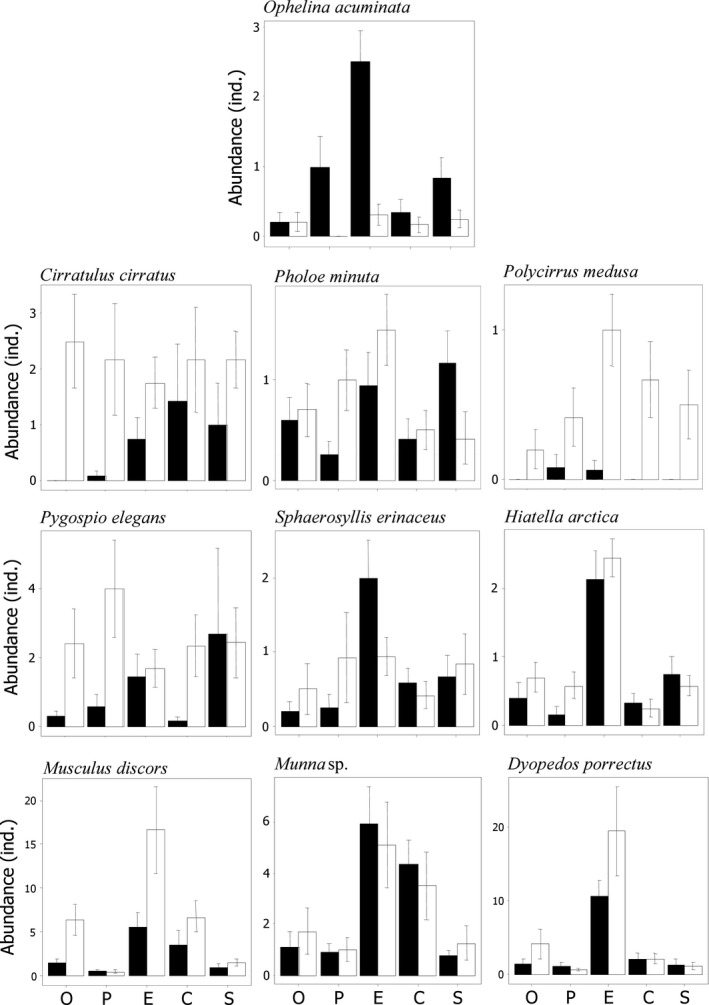
Mean (±*SE*) abundances by treatment of most abundant taxa affected by manipulations (significant Treatment, Live or Treatment × Live effects, see Tables 3 and 4) in the field experiment. C, crab enclosures; E, exclosure cages; O, open blocks; P, partial cages; S, shrimp enclosures; white bars denote live barnacles (LB), and black bars denote empty barnacle tests (ET)

Of 15 most abundant taxa, 10 responded to Treatment, Live, or their interaction (see Tables 2 and 3 for ANCOVA results). Polychaetes *Cirratulus cirratus*, *Polycirrus medusa*, *Pygospio elegans,* and a bivalve *Musculus discors* were significantly more abundant in LB than ET, whereas a polychaete *Ophelina acuminata* was significantly more abundant in ET than LB. Treatment affected the abundances of bivalves *Hiatella arctica* and *Musculus discors*, and an amphipod *Dyopedos porrectus*: they were significantly more abundant in predator exclosures than in any other treatments. Out of 600 *Musculus discors* and 116 *Hiatella arctica* individuals totally found, 597 and 108, correspondingly, were juveniles within 6 mm shell length. An isopod *Munna* sp. was also affected by Treatment, being equally abundant in crab cages and predator exclosures and significantly less abundant in open blocks, partial cages, and shrimp cages. While the polychaetes *Ophelina acuminata* and *Sphaerosyllis erinaceus* significantly responded to Treatment, their *post hoc* tests were inconclusive, although the abundance of both species was highest in predator exclosures. The results of *post hoc* tests and mean abundances by Live and Treatment levels are summarized in Table 3 and Figure 5.

## 4 DISCUSSION

Consumer control is not generally expected to determine community structure at high latitudes (Freestone et al., 2011; Schemske et al., 2009). Our experiments, however, show a surprisingly pronounced effect of epibenthic predators on mobile fauna of barnacle clusters. While predator removals had no impact on species diversity, they almost tripled total abundances both in LB and ET, strongly altered community structure, and increased the number of taxa per experimental unit. The effect of treatments was species‐specific with amphipods, isopods, and juvenile bivalves mostly affected by predators. Polychaetes, in contrast, essentially responded to the presence of live barnacles versus their empty tests rather than predator exclusion. In the presence of predators (i.e., in open blocks, partial, crab, and shrimp cages), assemblages associated with live barnacles and their empty tests grouped separately, whereas LB and ET from predator exclosures were relatively similar and differed from any other treatments. While LB and ET were significantly different in total abundance, abundance of several dominant taxa, species diversity, and multivariate community structure, none of the parameters analyzed were affected by Live × Treatment interaction (followed with consistent *post hoc* tests), showing that live barnacles and their empty tests similarly mitigate (or do not mitigate all) predator‐related effects.

Difference in assemblages associated with live barnacles and their empty tests is consistent with previous reports from this and other habitats (Barnes, 2000; Fairweather, 1990; Harley & O'Riley, 2011; Yakovis & Artemieva, 2015). Likewise, dead coral microhabitats have a different community structure of associated organisms compared with live colonies (Coles, 1980; Head et al., 2015; Leray, Beraud, Anker, Chancerelle, & Mills, 2012). Empty barnacle tests comprise more cavities and accumulate 2–3 times more sediment than live barnacles of the same size (Yakovis & Artemieva, 2015), which could provide better shelter from predation for at least some of the associated taxa. On the other hand, cirral activity of live barnacles could also interfere with predators resulting in lower predation pressure. Bleached and dead corals, for instance, provide poorer protection from predators for the associated damselfishes than live ones (Coker, Pratchett, & Munday, 2009). Despite the apparent presence of these potentially important mechanisms, the observed dissimilarity of mobile fauna associated with the two microhabitats does not noticeably result from predator‐related processes. Also, the impact of predator removal on mobile assemblages is much stronger than this dissimilarity. The taxa that exhibit higher abundances in LB may benefit from direct trophic facilitation by live barnacles (which alter local water flow by cirral movements and produce feces), like obligatory coral‐dwelling decapods consuming coral mucus (Head et al., 2015 and references therein).

All the taxa affected by predator presence manipulations were most abundant in exclosure cages. There are two ways predators can reduce the abundance of mobile prey: consumption and triggering predator‐avoidance behavior. Behavioral responses to the cues hinting potentially higher predation risks often make prey's “landscape of fear” an equally important driver of its spatial distribution compared with lethal attacks (Laundré, Hernández, & Altendorf, 2001; Preisser, Bolnick, & Bernard, 2005). The design of our experiments, however, is incapable of separating consumptive and nonconsumptive effects of predators. Consequently, the mechanisms behind the positive response to predator removal may vary between species depending on their mobility and predator preferential diet. While some flourish since they directly avoid consumption, others might immigrate to minimize predation risks. Suggesting which mechanism is more important for a particular prey species is complicated, because the diet of *Spirontocaris phippsii* and *Hyas araneus* in the White Sea is totally unexplored.

In fact, feeding habits of these two predators are generally obscure. According to stomach contents analysis, *Spirontocaris spinus* from an arctic fjord feeds on benthic foraminiferans and hydroids (Birkely & Gulliksen, 2003). Our unpublished laboratory experiments revealed that per day an individual *Spirontocaris phippsii* from the White Sea can consume tens to hundreds of small sessile invertebrates from various taxa (spirorbid worms, bryozoans, juvenile ascidians). Definitely a highly mobile omnivorous predator, yet *S. phippsii* has been never previously reported to forage on any mobile prey. Nitrogen isotopic signatures from northern Gulf of St. Lawrence (eastern Canada) show an estimated trophic level of *H. araneus* as 2.9 with *δ* ^15^N of 11.9 ± 0.1‰ (Nadon & Himmelman, 2010), which identifies it as a predator or scavenger. In contrast to other crustacean macropredators, *Hyas* spp. has no effect on species composition and abundances of mobile benthic fauna from unstructured subtidal soft sediments in Bonne Bay, Newfoundland (Quijón & Snelgrove, 2005). Few other sources report *Hyas araneus* as a scavenger (Nickell & Moore, 1991, 1992) or a predator on live juvenile sea scallops (Nadeau, Barbeau, & Brêthes, 2009; Nadeau & Cliche, 1998). All these scarce data come from the habitats where *Hyas* shares a guild with several other crab species. In the White Sea, though, with the exception of a single hermit crab species (which generally occurs deeper), *Hyas araneus* is an only crab found, which may broaden its food preferences.

Of top abundant taxa, all those clearly affected by predator presence were epibenthic crustaceans and juvenile bivalves, in contrast to mostly infaunal polychaetes. At our research sites near Solovetsky Islands, bivalves *Musculus discors* and *Hiatella arctica* apparently lose mobility as they become adult. Adult *M. discors* here are commonly associated with solitary ascidians, either embedded in ascidian tunic or within the nests made of byssal threads integrated into ascidian clumps attached to barnacle clusters (Yakovis & Artemieva, 2017; Yakovis et al., 2008). Adult *H. arctica* are mostly integrated into barnacle clusters, firmly anchored with byssal threads between barnacles or inside their empty tests (E. Yakovis and A. Artemieva, unpub. data). Our results suggest that mobile juveniles of both species constitute a relatively easy target for epibenthic predators, and finding suitable permanent refuges is their only chance to survive to adulthood. Interestingly, *Musculus discors* here acts as a secondary foundation species, since their nests frequently serve as a substrate for several species of red algae (Yakovis & Artemieva, 2017). Consequently, the availability of this resource for red algae and the suite of their epibionts is regulated by a trophic cascade. Although nonconsumptive effects of *Hyas* and *Spirontocaris* on juvenile *Musculus* and *Hiatella* are possible, it is more likely that these relatively slowly moving bivalves are directly consumed. At least, other shrimp and crab species frequently forage on mollusk juveniles and thus control the abundance of adults (Beukema, Honkoop, & Dekker, 1998; Eggleston, 1990).

An amphipod *Dyopedos porrectus*, which also exhibited a strong negative response to predator presence, moves faster than bivalve juveniles, but builds flexible rods 4–6 cm long attached to surfaces of hard substrates, on which it stays hooked when feeding on suspended particles (Mattson & Cedhagen, 1989; Thiel, 1999). Consistent with our results, a sand shrimp *Crangon septemspinosa* eliminates a similar rod‐building *Dyopedos monacanthus* in laboratory experiments (Thiel, 1999). Unlike *Dyopedos*, another abundant amphipod *Crassicorophium bonellii* is not affected by predator removals. *Crassicorophium*, however, is a tubicolous deposit‐suspension feeder dwelling inside an open‐ended tube it pumps water through to collect food particles (Foster‐Smith & Shillaker, 1977) which apparently helps this and some other corophiids (Mook, 1983) to evade predators. An isopod *Munna* sp. positively responded to predator removals, being solely affected by shrimp and not crabs. *Munna* is also epibenthic, usually found on the surfaces of hard substrates, and not a tube‐builder (Ambrose & Anderson, 1990; Hayward & Ryland, 2017). These isopods are extremely fast runners and look much more vigilant than *Dyopedos* in the laboratory (authors' personal observations), which may explain their invulnerability to relatively sluggish crabs.

In unstructured muddy sand and seagrass meadows, the removal of epibenthic predators causes the increase in abundance of infaunal predators which may lead to increased predation pressure on the rest of infauna (Ambrose, 1984). In our experiments, *Harmothoe imbricata*, *Pholoe minuta,* and *Sphaerosyllis erinaceus*, being surface carnivores (Ambrose, 1993; Plyuscheva, Martin, & Britaev, 2010), according to *post hoc* tests, were not significantly affected by manipulations. The increase of *Sphaerosyllis* abundance in predator exclosures, however, was only marginally insignificant (Tables 2 and 3). In the field, *Pholoe* and *Sphaerosyllis* inhabit barnacle clusters rather than surrounding muddy sand (Yakovis et al., 2005), while all the three species significantly increase their abundance in response to adding structure (PVC tubes) to unstructured sediment (Yakovis et al., 2007). Given that some crab species can prey at least on *Pholoe* (Quijón & Snelgrove, 2005), it is likely that structural traits of barnacle clusters effectively protect these potentially vulnerable mesopredators from crabs and shrimp compared with less structured habitats.

At global geographical scale, strength of biotic interactions, including predation, is commonly supposed to reduce with latitude (Schemske et al., 2009). For instance, predator removals have almost no effect on species richness and abundances in temperate (41° N) compared with tropical (9° N) sessile epibenthic assemblages developing on PVC settlement panels (Freestone et al., 2011). This pattern supposedly results from impact on species' evolution of higher environmental stress level closer to the poles (Schemske et al., 2009). Unexpectedly, predation is increasingly found important at high latitudes (Giachetti, Battini, Bortolus, Tatián, & Schwindt, 2019 and references therein). Highest intensity of predation is generally linked to low abiotic stress, and so are associational defenses from predators backed by strong facilitators such as FS (Bruno et al., 2003). Subtidal habitats, however, are obviously much less affected by abiotic stress than commonly studied intertidal ones, especially in harsh environment of polar and subpolar seas with winter ice cover. At our research site, even most severe fall storms apparently do not largely influence benthic communities at 12‐m‐deep seafloor, since rather fragile barnacle‐dominated epibenthic patches on empty bivalve shells can persist here for years (Varfolomeeva et al., 2008; Yakovis et al., 2005). This can cause local increase both in predation intensity and associational defenses by FS compared with the levels predicted solely from latitude. Consistently, many examples of strong trophic control in subpolar and polar waters come from subtidal habitats (Estes & Palmisano, 1974; Giachetti et al., 2019; Quijón & Snelgrove, 2005), although inter‐ and subtidal predation levels have been never specifically compared across latitudes.

It also appears that communities shaped by FS in general do not necessarily follow the latitudinal predation gradient rule. Seagrasses appear to be the only functional group of FS that span from tropical to polar waters studied extensively enough to allow a cross‐latitude comparison. Direct consumer control of the fauna associated with seagrass meadows rather shows the reverse latitude gradient, if any. Being relatively strong at 58° N (Moksnes, Gullström, Tryman, & Baden, 2008), 37° N (Douglass et al., 2007), and 30° N (Leber, 1985), it is weak or absent at 35° N (Summerson & Peterson, 1984) and 26° N (Hammerschlag‐Peyer et al., 2013). Although the areas covered by FS‐generated habitats may globally decrease with latitude, the predation strength within these islands of relative stability may not be primarily controlled by environmental stress, which is ameliorated by FS. Here, we show that even at 65° N predators can severely affect abundances (but not the diversity) of the fauna associated with FS. Relative strength of top‐down versus bottom‐up control in FS‐driven communities is often switched by human disturbance (Bertness et al., 2014). It is thus important that the White Sea has faced a dramatic decline of fishery in recent decades (Berger, 2005); hence, our results are unlikely biased by anthropogenic influence on apex predators.

## CONFLICT OF INTEREST

None declared.

## AUTHOR CONTRIBUTIONS

EY conceived and designed the experiments, performed the experiments, analyzed the data, contributed reagents/materials/analysis tools, wrote the paper, prepared figures and tables, and reviewed drafts of the paper. AA conceived and designed the experiments, performed the experiments, contributed reagents/materials/analysis tools, and reviewed drafts of the paper.

## Data Availability

Raw abundances of all the taxa identified in the experimental units upon retrieval: Dryad https://doi.org/10.5061/dryad.8jm0c3n.

## References

[ece35570-bib-0001] Almany, G. R. (2004). Differential effects of habitat complexity, predators and competitors on abundance of juvenile and adult coral reef fishes. Oecologia, 141, 105–113. 10.1007/s00442-004-1617-0 15197644

[ece35570-bib-0002] Almany, G. R. , & Webster, M. S. (2006). The predation gauntlet: Early post‐settlement mortality in reef fishes. Coral Reefs, 25, 19–22. 10.1007/s00338-005-0044-y

[ece35570-bib-0003] Ambrose, R. F. , & Anderson, T. W. (1990). Influence of an artificial reef on the surrounding infaunal community. Marine Biology, 107, 41–52. 10.1007/BF01313240

[ece35570-bib-0004] Ambrose, W. G., Jr. (1984). Role of predatory infauna in structuring marine soft‐bottom communities. Marine Ecology Progress Series, 17, 109–115. 10.3354/meps017109

[ece35570-bib-0005] Ambrose, W. G., Jr. (1993). Effects of predation and disturbance by ophiuroids on soft‐bottom community structure in Oslofjord: Results of a mesocosm study. Marine Ecology Progress Series, 97, 225–236. 10.3354/meps097225

[ece35570-bib-0006] Anderson, M. J. (2001). A new method for non‐parametric multivariate analysis of variance. Austral Ecology, 26, 32–46.

[ece35570-bib-0007] Anderson, M. J. (2006). Distance‐based tests for homogeneity of multivariate dispersions. Biometrics, 62, 245–253. 10.1111/j.1541-0420.2005.00440.x 16542252

[ece35570-bib-0008] Anderson, M. J. , Gorley, R. N. , & Clarke, K. R. (2008). PERMANOVA+ for PRIMER: Guide to software and statistical methods. Plymouth, UK: PRIMER‐E.

[ece35570-bib-0009] Anderson, M. J. , & Walsh, D. C. I. (2013). PERMANOVA, ANOSIM, and the Mantel test in the face of heterogeneous dispersions: What null hypothesis are you testing? Ecological Monographs, 83, 557–574. 10.1890/12-2010.1

[ece35570-bib-0010] Barnes, M. (2000). The use of intertidal barnacle shells. Oceanography and Marine Biology: An Annual Review, 38, 157–187.

[ece35570-bib-0011] Beets, J. (1997). Effects of a predatory fish on the recruitment and abundance of Caribbean coral reef fishes. Marine Ecology Progress Series, 148, 11–21. 10.3354/meps148011

[ece35570-bib-0012] Bell, J. D. , & Westoby, M. (1986). Abundance of macrofauna in dense seagrass is due to habitat preference, not predation. Oecologia, 68, 205–209. 10.1007/BF00384788 28310128

[ece35570-bib-0013] Berger, V. Y. (2005) Production potential and commercial poorness of the White Sea In Stogov I. A., Railkin A. I., & Levitin M. G. (Eds.), The 30th Anniversary of the SPSU MBS: results and prospects (pp. 7–24). St.‐Petersburg, Russia:St.-Petersburg State University [in Russian].

[ece35570-bib-0014] Bertness, M. D. , Brisson, C. P. , Coverdale, T. C. , Bevil, M. C. , Crotty, S. M. , & Suglia, E. R. (2014). Experimental predator removal causes rapid salt marsh die‐off. Ecology Letters, 17, 830–835. 10.1111/ele.12287 24766277 PMC4286111

[ece35570-bib-0015] Beukema, J. J. , Honkoop, P. J. C. , & Dekker, R. (1998). Recruitment in *Macoma balthica* after mild and cold winters and its possible control by egg production and shrimp predation. Hydrobiologia, 375(376), 23–34.

[ece35570-bib-0016] Birkely, S. R. , & Gulliksen, B. (2003). Feeding ecology in five shrimp species (Decapoda, Caridea) from an Arctic fjord (Isfjorden, Svalbard), with emphasis on *Sclerocrangon boreas* (Phipps, 1774). Crustaceana, 76, 699–715.

[ece35570-bib-0017] Boaden, A. E. , & Kingsford, M. J. (2015). Predators drive community structure in coral reef fish assemblages. Ecosphere, 6, 1–33.

[ece35570-bib-0018] Bruno, J. F. , Stachowicz, J. J. , & Bertness, M. D. (2003). Inclusion of facilitation into ecological theory. Trends in Ecology & Evolution, 18, 119–125. 10.1016/S0169-5347(02)00045-9

[ece35570-bib-0019] Caley, M. J. (1993). Predation, recruitment and the dynamics of communities of coral‐reef fishes. Marine Biology, 117, 33–43. 10.1007/BF00346423

[ece35570-bib-0020] Carr, M. H. , & Hixon, M. A. (1995). Predation effects on early post‐settlement survivorship of coral‐reef fishes. Marine Ecology Progress Series, 124, 31–42. 10.3354/meps124031

[ece35570-bib-0021] Clarke, K. R. (1993). Non‐parametric multivariate analyses of changes in community structure. Austral Ecology, 18, 117–143. 10.1111/j.1442-9993.1993.tb00438.x

[ece35570-bib-0022] Coker, D. J. , Pratchett, M. S. , & Munday, P. L. (2009). Coral bleaching and habitat degradation increase susceptibility to predation for coral‐dwelling fishes. Behavioral Ecology, 20, 1204–1210. 10.1093/beheco/arp113

[ece35570-bib-0023] Coles, S. L. (1980). Species diversity of decapods associated with living and dead reef coral *Pocillopora meandrina* . Marine Ecology Progress Series, 2, 281–291. 10.3354/meps002281

[ece35570-bib-0024] Douglass, J. G. , Duffy, J. E. , Spivak, A. C. , & Richardson, J. P. (2007). Nutrient versus consumer control of community structure in a Chesapeake Bay eelgrass habitat. Marine Ecology Progress Series, 348, 71–83. 10.3354/meps07091

[ece35570-bib-0025] Edgar, G. J. , & Aoki, M. (1993). Resource limitation and fish predation: Their importance to mobile epifauna associated with Japanese *Sargassum* . Oecologia, 95, 122–133. 10.1007/BF00649515 28313320

[ece35570-bib-0026] Eggleston, D. B. (1990). Foraging behavior of the blue crab, *Callinectes sapidus*, on juvenile oysters, *Crassostrea virginica*: Effects of prey density and size. Bulletin of Marine Science, 46, 62–82.

[ece35570-bib-0027] Eggleston, D. B. , Lipcius, R. N. , & Grover, J. J. (1997). Predator and shelter‐size effects on coral reef fish and spiny lobster prey. Marine Ecology Progress Series, 149, 43–59. 10.3354/meps149043

[ece35570-bib-0028] Estes, J. A. , & Palmisano, J. F. (1974). Sea otters: Their role in structuring nearshore communities. Science, 185, 1058–1060. 10.1126/science.185.4156.1058 17738247

[ece35570-bib-0029] Fairweather, P. G. (1990). Is predation capable of interacting with other community processes on rocky reefs? Austral Ecology, 15, 453–464. 10.1111/j.1442-9993.1990.tb01470.x

[ece35570-bib-0030] Foster, B. A. (1987). Barnacle ecology and adaptation In Southward A. J. (Ed.), Crustacean issues 5. Barnacle biology (pp. 113–134). Rotterdam, the Netherlands: AA Balkema.

[ece35570-bib-0031] Foster‐Smith, R. L. , & Shillaker, R. O. (1977). Tube irrigation by *Lembos websteri* Bate and *Corophium bonnelli* Milne Edwards (Crustacea: Amphipoda). Journal of Experimental Marine Biology and Ecology, 26, 289–296. 10.1016/0022-0981(77)90088-0

[ece35570-bib-0032] Freestone, A. L. , Osman, R. W. , Ruiz, G. M. , & Torchin, M. E. (2011). Stronger predation in the tropics shapes species richness patterns in marine communities. Ecology, 92, 983–993. 10.1890/09-2379.1 21661559

[ece35570-bib-0033] Giachetti, C. B. , Battini, N. , Bortolus, A. , Tatián, M. , & Schwindt, E. (2019). Macropredators as shapers of invaded fouling communities in a cold temperate port. Journal of Experimental Marine Biology and Ecology, 518, 151177 10.1016/j.jembe.2019.151177

[ece35570-bib-0034] Gil, M. A. , & Pfaller, J. B. (2016). Oceanic barnacles act as foundation species on plastic debris: Implications for marine dispersal. Scientific Reports, 6, 19987 10.1038/srep19987 26813348 PMC4728489

[ece35570-bib-0035] Hammerschlag‐Peyer, C. M. , Allgeier, J. E. , & Layman, C. A. (2013). Predator effects on faunal community composition in shallow seagrass beds of The Bahamas. Journal of Experimental Marine Biology and Ecology, 446, 282–290. 10.1016/j.jembe.2013.06.002

[ece35570-bib-0036] Harley, C. D. G. (2006). Effects of physical ecosystem engineering and herbivory on intertidal community structure. Marine Ecology Progress Series, 317, 29–39. 10.3354/meps317029

[ece35570-bib-0037] Harley, C. D. G. , & O'Riley, J. L. (2011). Non‐linear density‐dependent effects of an intertidal ecosystem engineer. Oecologia, 166, 531–541. 10.1007/s00442-010-1864-1 21170751

[ece35570-bib-0038] Hayward, P. J. , & Ryland, J. S. (2017). Handbook of the marine fauna of North‐West Europe, (2nd ed.). Oxford, UK: Oxford University Press.

[ece35570-bib-0039] Head, C. E. I. , Bonsall, M. B. , Koldewey, H. , Pratchett, M. S. , Speight, M. , & Rogers, A. D. (2015). High prevalence of obligate coral‐dwelling decapods on dead corals in the Chagos Archipelago, central Indian Ocean. Coral Reefs, 34, 905–915. 10.1007/s00338-015-1307-x

[ece35570-bib-0040] Heck, K. L. Jr , & Orth, R. J. (2006). Predation in seagrass beds In Larkum A. W. D., Orth R. J., & Duarte C. M. (Eds.), Seagrasses: Biology, ecology, and conservation (pp. 537–550). Dordrecht, the Netherlands: Springer.

[ece35570-bib-0041] Heinlein, J. M. , Stier, A. C. , & Steele, M. A. (2010). Predators reduce abundance and species richness of coral reef fish recruits via non‐selective predation. Coral Reefs, 29, 527–532. 10.1007/s00338-010-0592-7

[ece35570-bib-0042] Hixon, M. A. , & Beets, J. P. (1993). Predation, prey refuges, and the structure of coral‐reef fish assemblages. Ecological Monographs, 63, 77–101. 10.2307/2937124

[ece35570-bib-0043] Hughes, T. P. , Rodrigues, M. J. , Bellwood, D. R. , Ceccarelli, D. , Hoegh‐Guldberg, O. , McCook, L. , … Willis, B. (2007). Phase shifts, herbivory, and the resilience of coral reefs to climate change. Current Biology, 17, 360–365. 10.1016/j.cub.2006.12.049 17291763

[ece35570-bib-0044] Kayal, M. , Vercelloni, J. , Lison de Loma, T. , Bosserelle, P. , Chancerelle, Y. , Geoffroy, S. , … Adjeroud, M. (2012). Predator crown‐of‐thorns starfish (*Acanthaster planci*) outbreak, mass mortality of corals, and cascading effects on reef fish and benthic communities. PLoS ONE, 7, e47363 10.1371/journal.pone.0047363 23056635 PMC3466260

[ece35570-bib-0045] Laundré, J. W. , Hernández, L. , & Altendorf, K. B. (2001). Wolves, elk, and bison: Reestablishing the “landscape of fear” in Yellowstone National Park, U.S.A. Canadian Journal of Zoology, 79, 1401–1409. 10.1139/z01-094

[ece35570-bib-0046] Leber, K. M. (1985). The influence of predatory decapods, refuge, and microhabitat selection on seagrass communities. Ecology, 66, 1951–1964. 10.2307/2937391

[ece35570-bib-0047] Leray, M. , Beraud, M. , Anker, A. , Chancerelle, Y. , & Mills, S. C. (2012). *Acanthaster planci* outbreak: Decline in coral health, coral size structure modification and consequences for obligate decapod assemblages. PLoS ONE, 7, e35456 10.1371/journal.pone.0035456 22530026 PMC3328453

[ece35570-bib-0048] Lewis, S. M. (1986). The role of herbivorous fishes in the organization of a Caribbean reef community. Ecological Monographs, 56, 183–200. 10.2307/2937073

[ece35570-bib-0049] Mattson, S. , & Cedhagen, T. (1989). Aspects of the behaviour and ecology of *Dyopedos monacanthus* (Metzger) and *D. porrectus* Bate, with comparative notes on *Dulichia tubereulata* Boeck (Crustacea: Amphipoda: Podoceridae). Journal of Experimental Marine Biology and Ecology, 127, 253–272.

[ece35570-bib-0050] Moksnes, P.‐O. , Gullström, M. , Tryman, K. , & Baden, S. (2008). Trophic cascades in a temperate seagrass community. Oikos, 117, 763–777. 10.1111/j.0030-1299.2008.16521.x

[ece35570-bib-0051] Mook, D. (1983). Responses of common fouling organisms in the Indian River, Florida, to various predation and disturbance intensities. Estuaries, 6, 372–379. 10.2307/1351396

[ece35570-bib-0052] Mumby, P. J. , Dahlgren, C. P. , Harborne, A. R. , & Kappel, C. V. (2006). Fishing, trophic cascades, and the process of grazing on coral reefs. Science, 311, 98–101. 10.1126/science.1121129 16400152

[ece35570-bib-0053] Nadeau, M. , Barbeau, M. A. , & Brêthes, J. C. (2009). Behavioural mechanisms of sea stars (*Asterias vulgaris* Verrill and *Leptasterias polaris* Muller) and crabs (*Cancer irroratus* Say and *Hyas araneus* Linnaeus) preying on juvenile sea scallops (*Placopecten magellanicus* (Gmelin)), and procedural effects of scallop tethering. Journal of Experimental Marine Biology and Ecology, 374, 134–143.

[ece35570-bib-0054] Nadeau, M. , & Cliche, G. (1998). Predation of juvenile sea scallops (*Placopecten magellanicus*) by crabs (*Cancer irroratus* and *Hyas* sp.) and starfish (*Asterias vulgaris*, *Leptasterias polaris*, and *Crossaster papposus*). Journal of Shellfish Research, 17, 905–910.

[ece35570-bib-0055] Nadon, M.‐O. , & Himmelman, J. H. (2010). The structure of subtidal food webs in the northern Gulf of St. Lawrence, Canada, as revealed by the analysis of stable isotopes. Aquatic Living Resources, 23, 167–176. 10.1051/alr/2010010

[ece35570-bib-0056] Nickell, T. D. , & Moore, P. G. (1991). The behavioural ecology of epibenthic scavenging invertebrates in the Clyde Sea area: Field sampling using baited traps. Cahiers De Biologie Marine, 32(3), 353–370.

[ece35570-bib-0057] Nickell, T. D. , & Moore, P. G. (1992). The behavioural ecology of epibenthic scavenging invertebrates in the Clyde Sea area: Laboratory experiments on attractions to bait in moving water, underwater TV observation in situ and general conclusions. Journal of Experimental Marine Biology and Ecology, 159, 15–35. 10.1016/0022-0981(92)90255-9

[ece35570-bib-0058] Plyuscheva, M. , Martin, D. , & Britaev, T. A. (2010). Diet analyses of the scale‐worms *Lepidonotus squamatus* and *Harmothoe imbricata* (Polychaeta, Polynoidae) in the White Sea. Marine Biology Research, 6, 271–281.

[ece35570-bib-0059] Preisser, E. L. , Bolnick, D. I. , & Bernard, M. F. (2005). Scared to death? The effects of intimidation and consumption in predator–prey interactions. Ecology, 86, 501–509. 10.1890/04-0719

[ece35570-bib-0060] Quijón, P. A. , & Snelgrove, P. V. R. (2005). Differential regulatory roles of crustacean predators in a sub‐arctic, soft‐sediment system. Marine Ecology Progress Series, 285, 137–149. 10.3354/meps285137

[ece35570-bib-0061] Schemske, D. W. , Mittelbach, G. G. , Cornell, H. V. , Sobel, J. M. , & Roy, K. (2009). Is there a latitudinal gradient in the importance of biotic interactions. Annual Review of Ecology Evolution and Systematics, 40, 245–269. 10.1146/annurev.ecolsys.39.110707.173430

[ece35570-bib-0062] Schutte, V. G. W. , & Byers, J. E. (2017). Variation in a simple trait of mangrove roots governs predator access to, and assemblage composition of, epibiotic sponges. Marine Ecology Progress Series, 573, 15–23. 10.3354/meps12160

[ece35570-bib-0063] StatSoft, Inc (2007). STATISTICA (data analysis software system), version 8.0. Retrieved from http://www.statsoft.com

[ece35570-bib-0064] Summerson, H. C. , & Peterson, C. H. (1984). Role of predation in organizing benthic communities of a temperate‐zone seagrass bed. Marine Ecology Progress Series, 15, 63–77. 10.3354/meps015063

[ece35570-bib-0065] Thiel, M. (1999). Extended parental care in marine amphipods II. Maternal protection of juveniles from predation. Journal of Experimental Marine Biology and Ecology, 234, 235–253. 10.1016/S0022-0981(98)00150-6

[ece35570-bib-0066] Thompson, R. C. , Wilson, B. J. , Tobin, M. L. , Hill, A. S. , & Hawkins, S. J. (1996). Biologically generated habitat provision and diversity of rocky shore organisms at a hierarchy of spatial scales. Journal of Experimental Marine Biology and Ecology, 202, 73–84. 10.1016/0022-0981(96)00032-9

[ece35570-bib-0067] Varfolomeeva, M. , Artemieva, A. , Shunatova, N. , & Yakovis, E. (2008). Growth and survival of barnacles in presence of co‐dominating solitary ascidians: Growth ring analysis. Journal of Experimental Marine Biology and Ecology, 363, 42–47. 10.1016/j.jembe.2008.06.012

[ece35570-bib-0068] Yakovis, E. , & Artemieva, A. (2015). Bored to death: Community‐wide effect of predation on a foundation species in a low‐disturbance arctic subtidal system. PLoS ONE, 10, e0132973 10.1371/journal.pone.0132973 26186648 PMC4506121

[ece35570-bib-0069] Yakovis, E. , & Artemieva, A. (2017). Cockles, barnacles and ascidians compose a subtidal facilitation cascade with multiple hierarchical levels of foundation species. Scientific Reports, 7, 237 10.1038/s41598-017-00260-2 28331222 PMC5427999

[ece35570-bib-0070] Yakovis, E. L. , Artemieva, A. V. , Fokin, M. V. , Grishankov, A. V. , & Shunatova, N. N. (2005). Patches of barnacles and ascidians in soft bottoms: Associated motile fauna in relation to the surrounding assemblage. Journal of Experimental Marine Biology and Ecology, 327, 210–224. 10.1016/j.jembe.2005.06.015

[ece35570-bib-0071] Yakovis, E. L. , Artemieva, A. V. , Fokin, M. V. , Varfolomeeva, M. A. , & Shunatova, N. N. (2007). Effect of habitat architecture on mobile benthic macrofauna associated with patches of barnacles and ascidians. Marine Ecology Progress Series, 348, 117–124. 10.3354/meps07060

[ece35570-bib-0072] Yakovis, E. L. , Artemieva, A. V. , Fokin, M. V. , Varfolomeeva, M. A. , & Shunatova, N. N. (2013). Synchronous annual recruitment variation in barnacles and ascidians in the White Sea shallow subtidal 1999–2010. Hydrobiologia, 706, 69–79. 10.1007/s10750-012-1340-5

[ece35570-bib-0073] Yakovis, E. L. , Artemieva, A. V. , Shunatova, N. N. , & Varfolomeeva, M. A. (2008). Multiple foundation species shape benthic habitat islands. Oecologia, 155, 785–795. 10.1007/s00442-007-0945-2 18193291

